# Inhibition of STAT3 Signaling Pathway by Terphenyllin Suppresses Growth and Metastasis of Gastric Cancer

**DOI:** 10.3389/fphar.2022.870367

**Published:** 2022-03-25

**Authors:** Dehua Yu, Simin Qi, Xiaoqing Guan, Wenkai Yu, Xuefei Yu, Maohua Cai, Qinglin Li, Weiyi Wang, Weidong Zhang, Jiang-Jiang Qin

**Affiliations:** ^1^ College of Pharmaceutical Sciences, Zhejiang Chinese Medical University, Hangzhou, China; ^2^ The Cancer Hospital of the University of Chinese Academy of Sciences (Zhejiang Cancer Hospital), Institute of Basic Medicine and Cancer (IBMC), Chinese Academy of Sciences, Hangzhou, China; ^3^ Key Laboratory of Marine Biogenetic Resources, Third Institute of Oceanography, Ministry of Natural Resources, Xiamen, China; ^4^ Institute of Interdisciplinary Integrative Medicine Research and Shuguang Hospital, Shanghai University of Traditional Chinese Medicine, Shanghai, China; ^5^ School of Pharmacy, Second Military Medical University, Shanghai, China

**Keywords:** STAT3, inhibitor, natural product, gastric cancer, terphenyllin

## Abstract

Gastric cancer is a common type of malignant tumor with a relatively poor prognosis and presents a serious threat to global health. Signal Transducer and Activator of Transcription-3 (STAT3) has been strongly implicated in many cancers, and its constitutive activation promotes growth, angiogenesis, inflammation, and immune evasion. Therefore, considerable efforts have been put into developing effective and safe STAT3 inhibitors. In this study, we performed a virtual screening by molecular docking and found that terphenyllin, a marine-derived natural product, directly interacted with STAT3. We further found that terphenyllin inhibited the phosphorylation and activation of STAT3 and decreased the protein levels of STAT3-dependent target genes, including c-Myc and Cyclin D1. Subsequently, we demonstrated that terphenyllin exerted its potent anticancer efficacy against gastric cancer *in vitro* and *in vivo*. Terphenyllin concentration-dependently inhibited growth, proliferation, and colony formation and induced cell cycle arrest and apoptosis of gastric cancer cells *in vitro*. Moreover, terphenyllin treatment suppressed the tumor growth and metastasis in a gastric cancer orthotopic mouse model without notable toxicity *in vivo*. Taken together, our results indicated that terphenyllin exerts its anticancer activity by inhibiting the STAT3 signaling pathway and may serve as a potent STAT3 inhibitor for gastric cancer treatment.

## Introduction

As a malignant tumor occupying a high incidence rate and mortality rate in the world, the existence of gastric cancer threatens human health. Early gastric cancer is generally asymptomatic, about 2/3 of gastric cancer is in the advanced stage at the time of initial diagnosis (Smyth et al., 2020). Even after radical resection, many patients will still suffer from local recurrence or distant metastasis. The 5-year survival rate in patients with gastric cancer is often less than 35%. A common cure for advanced gastric cancer is chemoradiotherapy. Most of the existing chemotherapy drugs have severe adverse reactions and high drug resistance, and it is often difficult for gastric cancer patients who fail the first-line and second-line chemotherapy to benefit from radiotherapy since radiotherapy is only a local treatment (Joshi and Badgwell, 2021). Molecular targeted therapy has played a considerable role in the treatment of advanced gastric cancer, while there are very limited targeted drugs that can be used in clinical treatment (Nakamura et al., 2021). The development of target drugs with high efficiency and low toxicity to gastric cancer is urgent for gastric cancer patients all over the world.

The signal transducer and activator of transcription (STAT) family of proteins are cytoplasmic transcription factors, which are responsible for the transmission of extracellular signals from the cell surface to the nucleus and activate gene transcription. STAT protein family (STAT1, STAT2, STAT3, STAT4, STAT5A, STAT5B, STAT6) has six common domains: amino-terminal domain, coiled-coil domain (CCD), DNA binding domain (DBD), linker domain (LD), SRC homologous domain (SH2) and carboxyl-terminal transactivation domain. Each domain has its specific physiological functions and mediates a variety of intracellular signal pathways (Tas et al., 2016). Among all STAT protein family members, STAT3 expression is required for some normal tissues and organs, such as bone marrow, peripheral nervous system, and digestive tract. In most human cancers, over-activation of STAT3 is usually associated with poor clinical prognosis. The transcriptional activity of STAT3 is involved in many biological processes, including cell proliferation, survival, angiogenesis, and immune evasion.

The abnormal activation of classical STAT3 mainly forms dimer through the combination of cytokines such as IL-6 and their corresponding cell surface receptors, thus triggering tyrosine phosphorylation cascade and then in turn recruiting glycoprotein 130 (gp130) and Janus kinases (JAKs), to phosphorylate and activate JAKs. On the contrary, JAK activation phosphorylates specific tyrosine residues of the receptor, followed by the interaction with the SH2 domain of STAT3, resulting in JAKs phosphorylating STAT3 in Tyr705. Phosphorylated STAT3 (p-STAT3) further forms homodimer by the binding between its phosphorylated Tyr705 site and SH2 domain, triggering the dissociation of STAT3 dimer from cell surface receptor and the translocation of STAT3 from the cytoplasm to the nucleus and further regulating downstream gene expression (Morris et al., 2018). Since the STAT3 signaling pathway has multiple influence mechanisms in tumor proliferation, metastasis, microenvironment formation, and immunosuppression, it has become a potential anti-tumor target (J. J. Qin et al., 2019).

**FIGURE 1:**
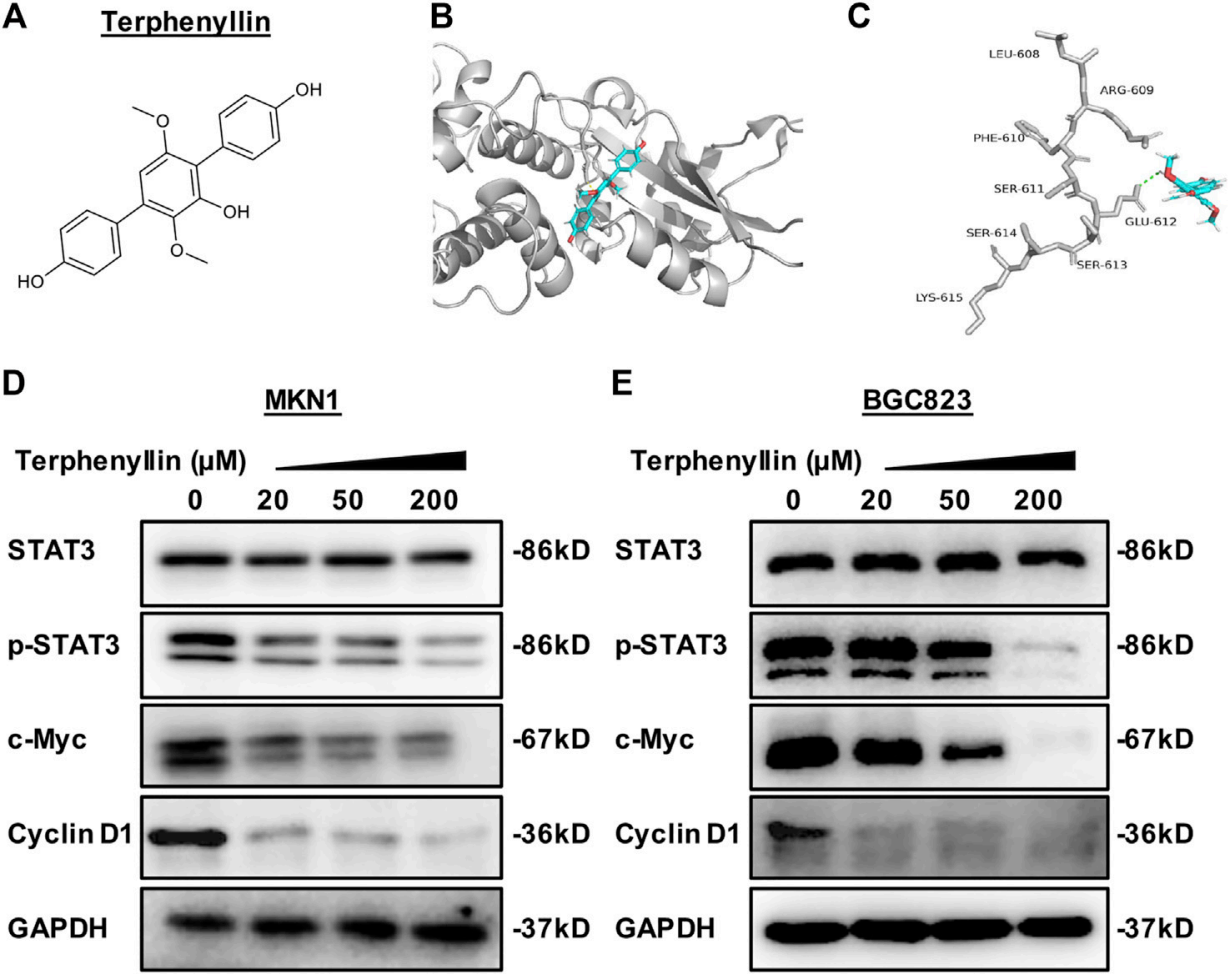
Terphenyllin binds to STAT3 to reduce the expression level of p-STAT3 and inhibit the STAT3 signaling pathway. **(A)** The chemical structure of terphenyllin. **(B,C)** The proposed binding mode of terphenyllin and STAT3. **(D,E)** MKN1 and BGC823 cells were treated with terphenyllin at the specified concentration for 24 h, and the levels of various proteins were detected by western blot analysis.

The development of efficient and specific small molecule inhibitors of the STAT3 signaling pathway has been challenged and much debated. Natural products provide abundant compounds with unique structures and exhibit a variety of remarkable biological activities, such as antibacterial, antifungal, and anticancer (Davison and Brimble, 2019; J.; Qin et al., 2017). Marine fungi have been proved to be a wide source of bioactive candidate drugs with novel structure and synthetic value (Jia et al., 2015; Haider et al., 2020). Over the past decade, we launched a project to identify new active compounds with anticancer efficacy from medicinal plants and marine fungi (Chen et al., 2013; Wang et al., 2018a). Indeed, through a high-through virtual screening based on structure, we identified a potential STAT3 inhibitor, 3ʼ,6ʼ-dimethoxy-1,1ʼ:4ʼ,1ʼʼ-terphenyl-2ʼ,4,4ʼʼ-triol, named terphenyllin (Figure 1A) from coral derived fungi. However, it is still not clear whether terphenyllin has anticancer efficacy in gastric cancer.

The purpose of this study was to evaluate the anticancer efficacy of terphenyllin *in vitro* and *in vivo* and its potential mechanism of action. Our results prove the potential of terphenyllin as a STAT3 inhibitor in the treatment of gastric cancer, which will provide a basis for the further development of this natural compound as a targeted drug.

## Materials and Methods

### Molecular Docking

The interaction between terphenyllin and STAT3 was investigated by molecular docking in this study. The structure of terphenyllin was drawn by ChemDraw 20.0. The crystal structure of STAT3 (PDB ID: 6TLC) was obtained from the RCSB Protein Data Bank (PDB, https://www.rcsb.org/). The binding sites between terphenyllin and STAT3 were predicted by AutoDock 4.2 software and the docking result was visualized by PyMol 2.5.

### Cell Lines and Chemicals

Human gastric cancer cell lines MKN1 and BGC823 were purchased from the American-type culture collection (ATCC). Both cell lines were cultured in RPMI 1640 medium at 37°C in an incubator containing 5% CO_2_. 10% fetal bovine serum (FBS) and 1% penicillin/streptomycin were added to cell culture media. The isolation and purification of terphenyllin were performed as previously (Wang et al., 2017). The structure of terphenylin was tested and verified by NMR, MS, UV, and IR spectroscopy. The purity of this compound was determined to be more than 98%. All antibodies were purchased from Cell Signaling Technology: Anti-STAT3 (Cat. No. 12640), Anti-pSTAT3 (Cat. No. 9145), Anti-c-Myc (Cat. No. 18583), Anti-Cyclin D1 (Cat. No. 55506), and Anti-GAPDH (Cat. No. 5174).

### Cell Viability, Colony Formation, Wound Healing, and Transwell Invasion Assays

The methodology used here was described elsewhere (Wang et al., 2014a; Wang et al., 2018b). To test the cytotoxic effect of terphenyllin, we seeded 3,000 cells in each well of 96-well plates. The compound was given at the indicated concentrations for 24, 48, and 72 h, followed by a CCK8 assay (Biosharp, Anhui, China). For colony formation assay, we seeded 800 cells in each well of 6-well plates and treated cells with the compound at the specific concentrations for 24 h. The cells were kept in the plate for 7–10 days, followed by fixation and crystal violet staining. In the wound healing assay, we passaged enough cells and formed a confluent monolayer of cells. The cells were scratched using a pipette tip and then exposed to the indicated concentrations of terphenyllin, thus assessing the effects of terphenyllin on cell migration. Considering the effect of terphenyllin on cell invasion, 5 × 10^4^ cells were transferred to every upper well of a Boyden chamber (Corning, United States) and treated with terphenyllin for 24 h. The invaded cells were then stained with Crystal Violet Stain solution 2.5% (Solarbio, China) and the stained cells were photographed and counted.

### Cell Cycle Assay and Apoptosis Assay

Cell cycle assay and apoptosis assay were performed as described previously (Wang et al., 2014b; Wang et al., 2019). For cell cycle assay, the 3×10^5^ cells were passaged to each well of 6-well plates (3×10^5^ cells/well) and exposed to indicated concentrations of terphenyllin (0, 20, 50, and 200 μM) for 24 h. The cells were harvested and fixed in 95% ethanol at 4 °C for more than 2 h. The fixed cells were incubated and stained with propidium iodide/RNase staining buffer (BD Pharmingen, United States). Subsequently, the cells in different cell cycle phases were analyzed by LSRFortessa (BD Bioscience, United States). The data were analyzed by the ModFit LT software (Verity Software House, Switzerland). For apoptosis assay, 3 × 10^5^ cells were seeded into 6-well plates and then treated with specific concentrations of terphenyllin for 48 h. The treated cells were harvested, washed with PBS, and then re-suspended in binding buffer and Annexin V-FITC and propidium iodide from FITC Annexin V Apoptosis Detection Kit I (BD Pharmingen, United States). The inhibitory effects of terphenyllin on cell apoptosis were processed on a CytoFLEX LX (Beckman Coulter, United States).

### Western Blot

Western blot was performed as described previously (Xue et al., 2017; J. J.; Qin et al., 2018). In brief, 3 × 10^5^ cells were seeded into 6-cm dishes and then treated with indicated concentrations of terphenyllin (0, 20, 50, and 200 μM) for 24 h. Subsequently, the cells were scraped and lysed in RIPA lysis buffer (Absin Bioscience Inc., Shanghai, China) containing protease inhibitor mixture (PMSF). After centrifugation for 20 min at 12,000 rpm, 4°C, the supernatants were collected. The concentration of the samples was then quantified by the BCA Protein Assay Kit (Absin, Shanghai, China). Equal amounts of protein were separated by 12% sodium dodecyl sulfate-polyacrylamide gel electrophoresis (SDS-PAGE) and then transferred onto nitrocellulose membranes (Bio-Rad, Hercules, CA, United States). The membranes were blocked with 5% milk for 2 h at room temperature. The membranes were washed three times with TBST (Tris-buffered saline, 0.1% Tween 20), followed by incubation with required primary antibodies overnight at 4°C. After washing three times with TBST, the membranes were incubated with a secondary antibody for 2 h at room temperature. The working dilution of each antibody involved in Western blot experiments is 1:1000. The membranes were scanned and visualized using the ImageQuant 800 (Amersham, United Kingdom).

### MKN1 Orthotopic Gastric Cancer Model

The orthotopic gastric cancer mouse model was formed as described previously (Kang et al., 2021). Female four or 5 weeks nude mice were purchased from Shanghai Laboratory Animal Center. In brief, 100 µL of MKN1-Luc cell solution (5 × 10^6^ cells in a 1:1 mixture of Matrigel and serum-free medium) was slowly injected into the left axillary. When the diameter of the MKN1 subcutaneous tumor grew to 1 cm, we took out the tumor and removed the fat and necrotic tissue of the tumor. Then we cut the fresh tumor tissue into a side length of 1-2 mm that were placed on the side of the great curvature of the stomach. Fifteen seconds later, we put the stomach back into the abdominal cavity, put a little powder antibiotic into the abdominal cavity, and suture the abdominal muscle layer and skin layer in turn. Terphenyllin was dissolved in PEG400:ethanol:saline (4:1:2, v/v/v) and administered to mice by intraperitoneal injection at a dose of 10 mg/kg/day, 7 days/week for 5 weeks. For *in vivo* imaging, mice were administered intraperitoneally with fluorescein substrate (150 mg/kg) and anesthetized with isoflurane using an anesthesia machine (Summit Anesthesia, United States). The images for orthotopic tumor growth and metastasis were detected from a Xenogen IVIS 200 imaging system (Caliper Life Sciences, United States). All the animal data was processed using LT Living Image 4.3 Software. At last, we tested the tumor metastasis to other organs in every experimental rice.

### Hematoxylin and Eosin (H&E) Staining

The H&E staining followed the previous paper (Voruganti et al., 2015). At the end of the animal experiments, various major organs including liver, lungs, kidneys, spleen, heart, and brain, were removed from the tumor-bearing mice, fixed in 10% formalin, and embedded in paraffin. These tissue blocks were handled and sectioned at a thickness of 5 µm. These tissue sections were deparaffinized with xylenes, rehydrated, washed using PBS, stained in Mayer’s Hematoxylin for 10 min, and then stained with eosin for less than 1 min. Following staining, the slides were dehydrated, mounted, and detected using an inverted microscope (Axio Observer A1, Zeiss, Germany).

### Statistical Analysis

Data were presented as the mean ± SD from three independent experiments and processed with the Prism software version 5 (Graph Pad Software Inc., San Diego, CA, United States). The significant differences between the two groups were compared by *t*-test. ∗ denotes *p* < 0.05, ∗∗ denotes *p* < 0.01, ∗∗∗ denotes *p* < 0.001.

## Results

### Terphenyllin Directly Binds to STAT3 and Suppresses STAT3 Activation in Gastric Cancer Cells

To identify small molecule inhibitors for STAT3, we performed a molecular docking and structure-based virtual screening assay against a library of natural products using STAT3 as a substrate, and terphenyllin was found to bind to STAT3. As shown in Figures 1B, C, the hydrogen atom of the phenolic hydroxyl group of terphenyllin forms a hydrogen bond with the oxygen atom of Glu612 side-chain amide. To investigate the effect of terphenyllin on the STAT3 signaling pathway, we test the inhibitory effect of terphenyllin on STAT3 protein in gastric cancer cells. We found that terphenyllin concentration-dependently suppressed the protein level of p-STAT3 (Tyr705) but had no obvious influence on the level of total STAT3 (Figures 1D, E). Also, terphenyllin inhibits the expression levels of Cyclin D1 and c-Myc, the downstream target genes of STAT3 (Figures 1D, E). In conclusion, the results above revealed that terphenyllin blocked the STAT3 signaling pathway in both MKN1 and BGC823 gastric cancer cell lines.

### Terphenyllin Inhibits Gastric Cancer Cell Proliferation and Colony Formation

**FIGURE 2:**
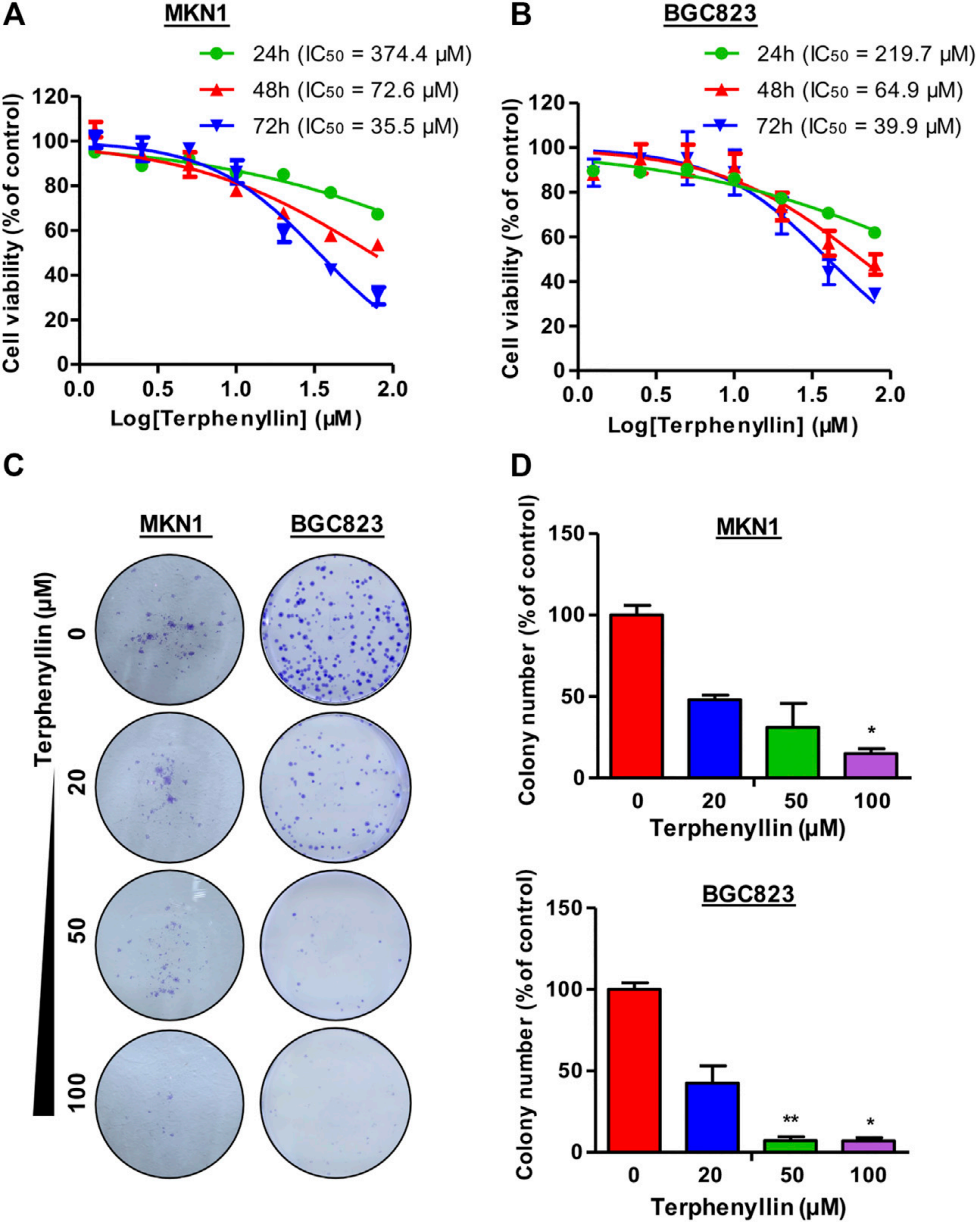
Terphenyllin inhibits gastric cancer cell growth *in vitro*. **(A,B)** 24, 48, 72 h for the cell viability assay to determine the 50% inhibitory concentration (IC50) values, followed by CCK8 assays. CCK8 experimental setup was repeated three times in parallel. **(C,D)** 24 h for the colony formation assay. Colony formation setup was repeated two times in parallel. Results are presented as mean ± SD. (∗*p* < 0.05; ∗∗*p* < 0.01, ∗∗∗*p* < 0.001).

CCK8 assay was performed to examine the effect of terphenyllin on the viability of gastric cancer cells. The results demonstrated that terphenyllin showed obvious inhibitory effects on the viability of gastric cancer cells especially when the treatment concentration was higher than 100 μM (Figures 2A, B). When the treatment time reached 72 h, the IC_50_ of terphenyllin against MKN1 and BGC823 cell lines reduced to 35.5 and 39.9 μM, respectively. We also explored the anticancer activity of terphenyllin using colony formation assay. Terphenyllin decreased the colony numbers of MKN1 and BGC823 cell lines in a concentration-dependent manner (Figures 2C, D). When the treatment concentration came to 20 μM, already inhibited by terphenyllin. We could hardly see any colony formation in both 2 cell lines at the highest concentration.

### Terphenyllin Induces Gastric Cancer Cell Apoptosis and Cell Cycle Arrest *in Vitro*

**FIGURE 3:**
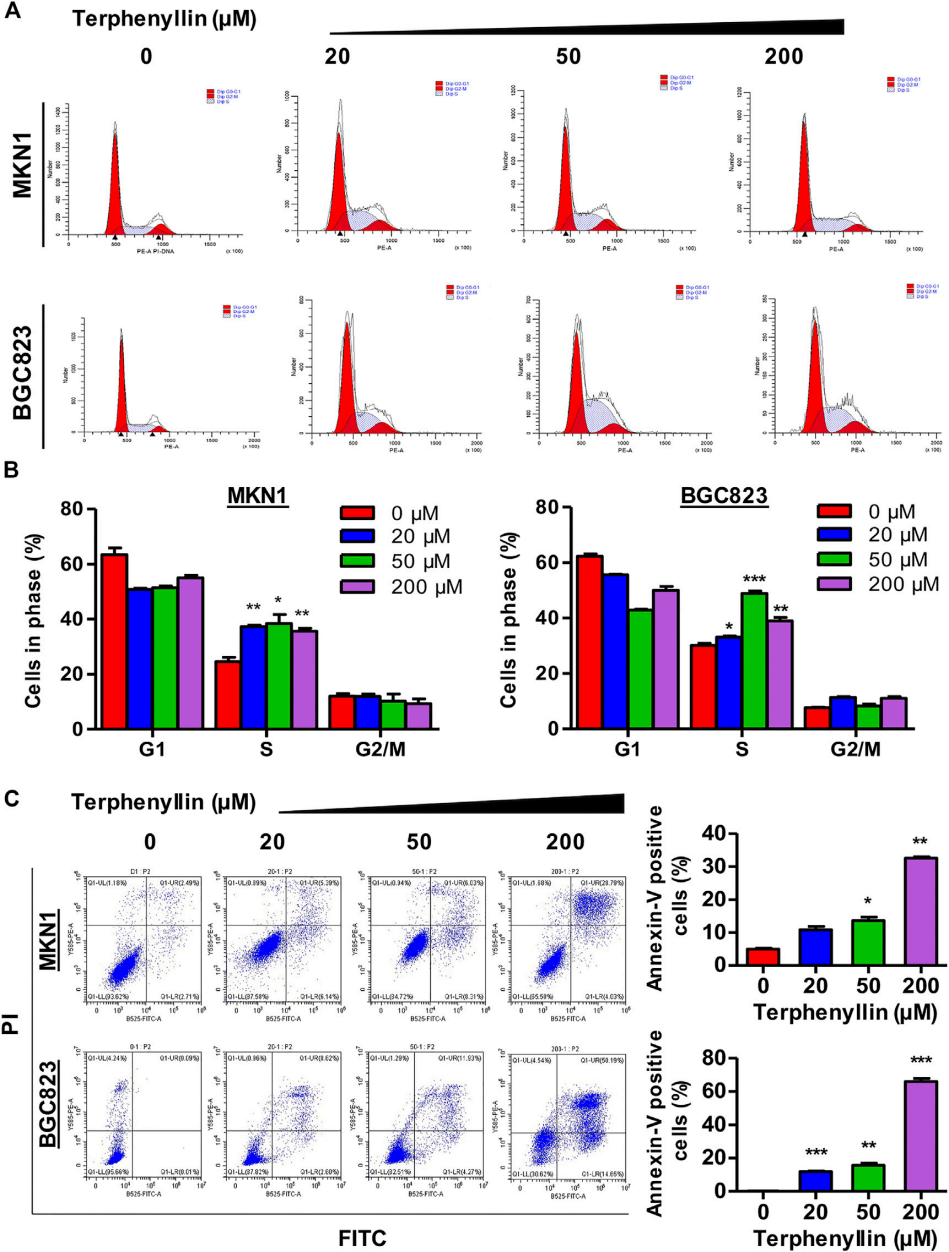
Terphenyllin can block the cell cycle of gastric cancer and induce apoptosis *in vitro.* **(A,B)** Terphenyllin was treated for 24 h and fixed, followed by the propidium iodide/RNase analysis. **(C)** MKN1 and BGC823 cells were treated with terphenyllin at the indicated concentrations for 48 h, followed by the detection of apoptosis by FITC-Annexin V assay. Results are presented as mean ± SD. (∗*p* < 0.05; ∗∗*p* < 0.01, ∗∗∗*p* < 0.001).

To test the effect of terphenyllin on the cell cycle progression, the cell cycle distribution of both MKN1 and BGC823 cell lines was measured by flow cytometric assay. It was seen that the proportions of G1 and G2 cells did not show remarkable changes among these four groups but the proportions of terphenyllin-treated groups in the S phase significantly increased, compared with the DMSO groups (Figures 3A, B). Terphenyllin treatment-induced cell cycle arrest was probably related to the down-regulation of cell cycle regulation proteins (such as Cyclin D1 and c-Myc), which was consistent with our western blot results. As for the assessment of the pro-apoptotic influence of terphenyllin on both cell lines, the apoptotic rates were detected by the FITC Annexin V apoptosis detection kit. As shown in Figure 3C, the proportion of apoptotic cells significantly increased in a concentration-dependent manner. In the highest concentration group, the percentage of apoptotic cells in both 2 cell lines was around 50%. Furthermore, it could be discovered that the apoptotic rate of BGC823 is much higher than that of MKN1, which implied that BGC823 cells might be more sensitive to terphenyllin than MKN1 cells.

### Terphenyllin Inhibits the Migration and Invasion of Gastric Cancer Cells *in Vitro*

**FIGURE 4:**
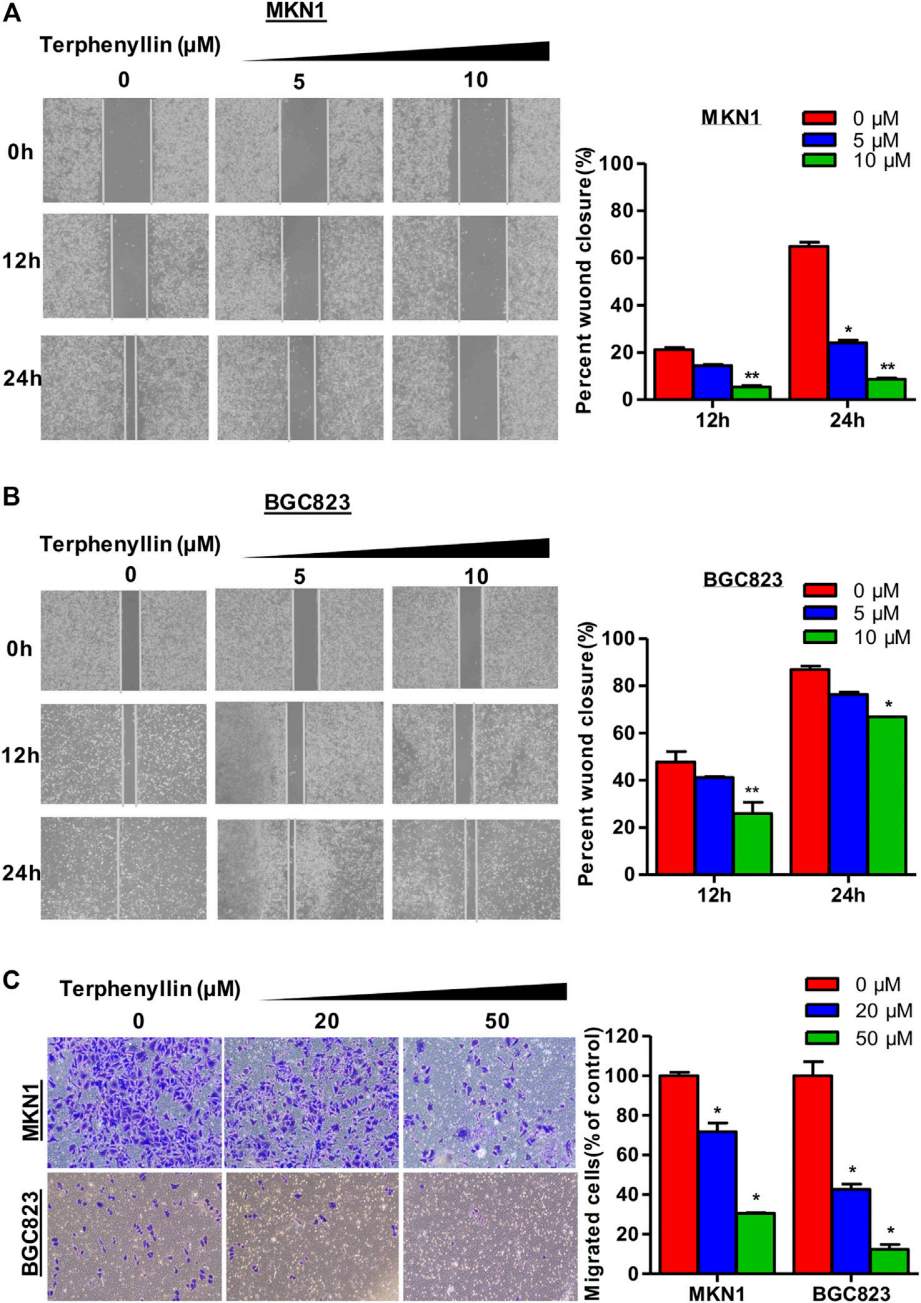
Terphenyllin prevented the invasion and metastasis of gastric cancer cells *in vitro*. **(A,B)** In the wound-healing assay, MKN1 and BGC823 cells were treated with terphenyllin at the specified concentration for 24 h, and the degree of cell healing was observed and recorded every 12 h. **(C)** Transwell invasion assay was carried out in MKN1 and BGC823 cells treated with or without terphenyllin. Results are presented as mean ± SD. (∗*p* < 0.05; ∗∗*p* < 0.01, ∗∗∗*p* < 0.001).

To determine the effects of terphenyllin on the migration and invasion of MKN1 and BGC823 cell lines, the wound-healing assay and transwell invasion assay were performed. As shown in Figures 4A, B, MKN1 and BGC823 cells in the control group both migrated into the entire wounded area by 48 h, whereas treatment with terphenyllin at specific concentrations (5 and 10 μM) prominently inhibited the cell migration. It was observed that the wound of the MKN1 cells was much easier to heal than of the BGC823 cells at a 10 μM treatment concentration. The results of the invasion assay in Figure 4C showed that compared with the control group, the invasive ability of MKN1 and BGC823 cells in the terphenyllin-treated groups was significantly decreased in a dose-dependent manner. Compared with BGC823 cells, MKN1 cells showed stronger invasive ability according to the number of cells invaded.

### Terphenyllin Suppresses Tumor Growth in the MKN1 Orthotopic Gastric Cancer Mouse Model

**FIGURE 5:**
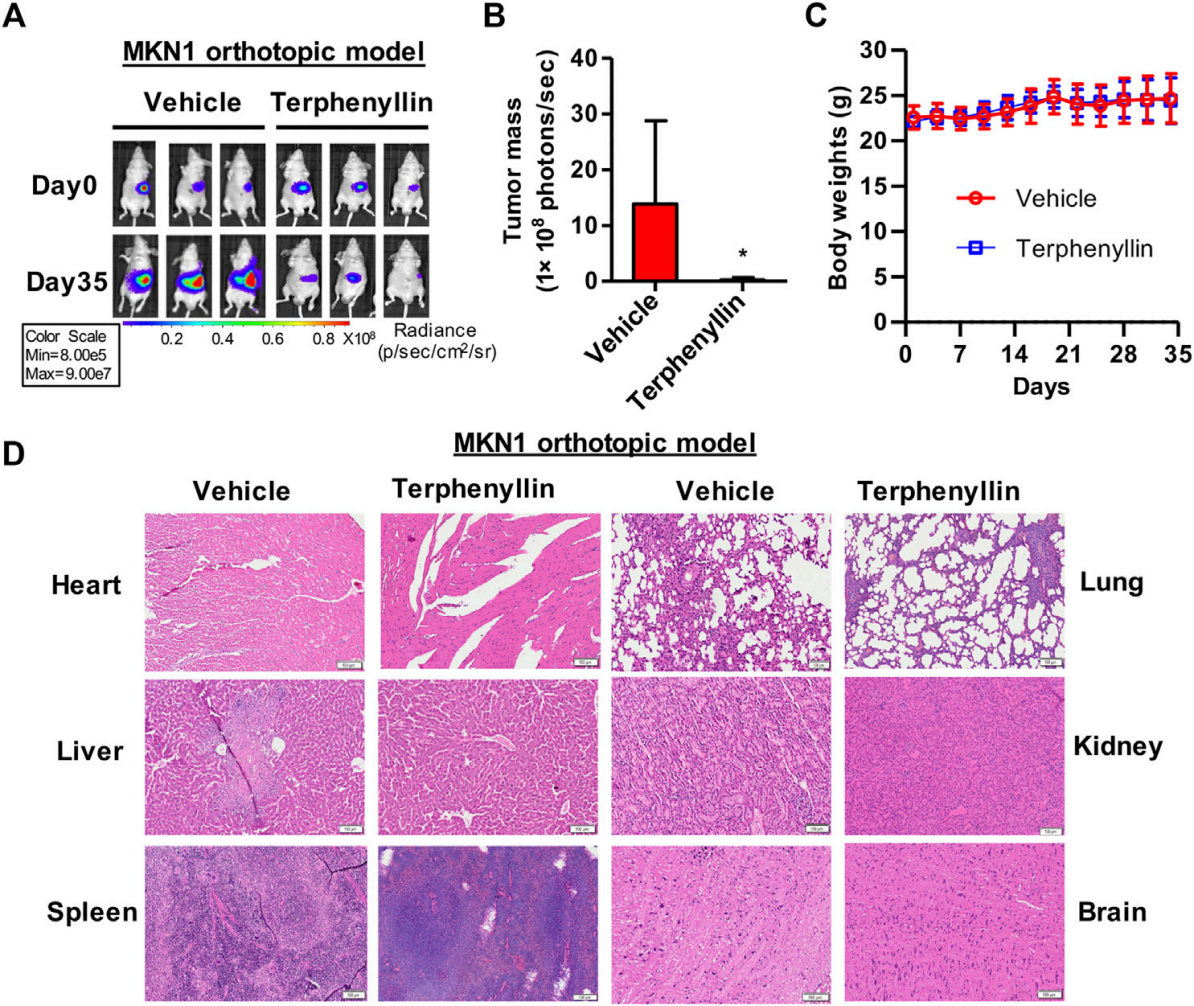
Terphenyllin suppresses the growth of MKN1 orthotopic tumors without causing any host toxicity. **(A)** The luciferase signal of MKN1 in model mice was detected every 7 days. **(B)** At the end of the experiment, the average tumor mass (determined by the detected photons/second) of terphenyllin treated mice and control mice was compared. **(C)** The weight of mice in the control group and the treated group was monitored every 3 days. **(D)** At the end of the experiment, samples were taken from the heart, liver, spleen, lung, kidney, and brain of mice, and the paraffin sections of these tissues were stained with H&E (all images represent sequence images) sections; scale bar, 100 µm. Results are presented as mean ± SD. (∗*p* < 0.05; ∗∗*p* < 0.01, ∗∗∗*p* < 0.001).

Owing to the inhibition of proliferation of gastric cancer cells after treatment with terphenyllin, we next tried to examine whether it could slow the tumor growth *in vivo*. We established an orthotopic gastric cancer mouse model and the tumor-bearing nude mice were randomly divided into two groups: vehicle group and terphenyllin treatment group. As expected, intraperitoneal injection of terphenyllin caused significant inhibition of gastric tumor growth in the orthotopic model (Figures 5A,B). The rate of tumor growth inhibition is 67.2% compared with the vehicle group. There was no significant difference in body weight between these two groups, revealing that terphenyllin had no obvious host toxicity (Figure 5C). The pathological changes in the vital organs were also evaluated using H&E staining. Compared the vehicle group with the treatment group, no pathological changes were detected, indicating that terphenyllin did not cause damage to the organs of mice (Figure 5D). Collectively, terphenyllin suppressed the growth of tumors and did not show significant side effects *in vivo*.

### Terphenyllin Prevents Tumor Metastasis in the MKN1 Orthotopic Gastric Cancer Mouse Model

**FIGURE 6:**
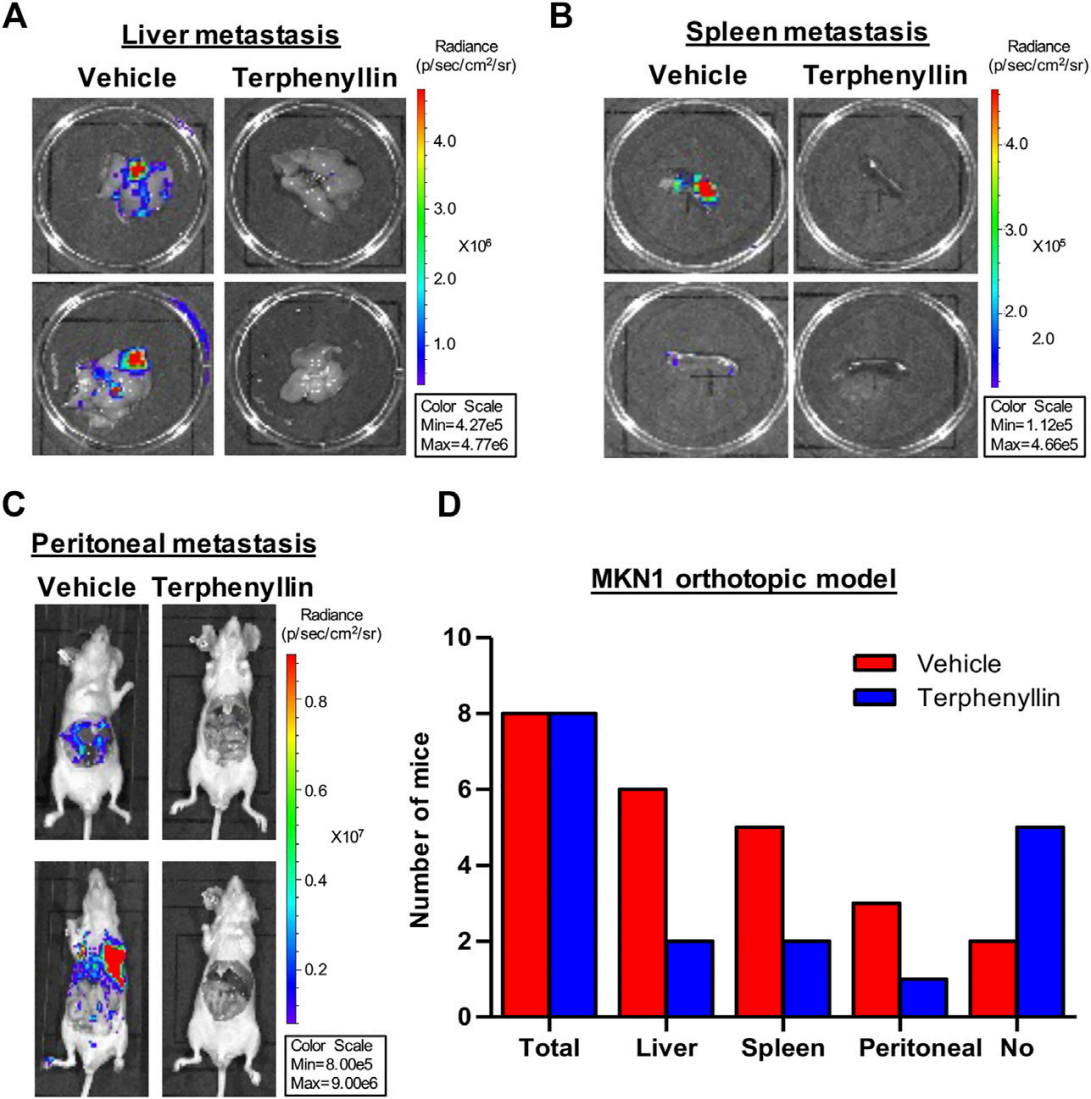
Terphenyllin inhibits gastric cancer metastasis *in vivo*. **(A,B)** At the end of the experiment of the MKN1 model, the liver and spleen were removed from mice and imaged to detect metastatic lesions. **(C)** The peritoneal cavity of mice was imaged to detect peritoneal metastasis. **(D)** The number of mice with liver, spleen, and peritoneal metastasis in the vehicle group and terphenyllin treated group was counted respectively.

To test the effect of terphenyllin on tumor metastasis, vital organs were taken out separately for fluorescence imaging. Terphenyllin showed an obvious inhibitory effect on the metastatic lesions in the liver, spleen, and peritoneum while comparing with the vehicle group. The statistical results indicated that 6, 2, and six out of eight vehicle-treated mice showed metastatic lesions in the liver, spleen and peritoneum, respectively, while the incidence of liver, spleen and peritoneal metastasis in terphenyllin-treated mice was reduced to 2/8, 1/8, and 2/8, respectively (Figures 6A–C). Moreover, there were far more mice without metastasis in the terphenyllin-treated group than in the vehicle group (Figure 6C).

## Discussion

Given that STAT3 is a potential target for cancer therapy, there are numerous direct and indirect STAT3 inhibitors. Indirect inhibitors interact with an upstream protein of the STAT3 signaling pathway that disturbs STAT3 by reducing the phosphorylation of STAT3 (Tyr705). Trienomycin A blocks the phosphorylation of STAT3, thus inhibiting the STAT3 pathway and exerting its anti-cancer therapy *in vitro* and *in vivo* (He et al., 2021). One of the major upstream targeting proteins is JAK kinase and a few JAK inhibitors have been carried out in some clinical trials. It is worth mentioning that ruxolitinib and tofacitinib have gained the approval of the US Food and Drug Administration. Some STAT3 inhibitors are designed to directly bind to the SH2 domain of STAT3 protein. The phosphopeptide is the first inhibitor on the SH2 domain of STAT3. Because of its poor stability and membrane permeability, recent studies mainly focus on identifying small molecule inhibitors of STAT3. A typical SH2 inhibitor C188-9 has been evaluated in gastric cancer and lung cancer, aiming at the development of advanced cancer therapy (Bharadwaj et al., 2016; Jung et al., 2017). OPB-111077 is another well-known inhibitor and has stepped into clinical experiments in some types of cancers (Genini et al., 2017). However, to our knowledge, there is still no STAT3 inhibitor approved to be used in the clinic.

In this study, we identified terphenyllin as a potential STAT3 inhibitor from coral-derived fungi through virtual structural-based screening. Recent studies have reported the extraction, isolation and structural analysis of terphenyllin (Singh et al., 2003; X. Q.; Zhang et al., 2018). There are a few preliminary studies that have demonstrated that terphenyllin and its analogs had significant cytotoxic effects on the lung, pancreatic cancer cells, and hepatoma cells (Wang et al., 2017; J.; Zhang et al., 2020; Haider et al., 2020). Terphenyllin induced the apoptosis of pancreatic cancer cell lines by modulating the expression of apoptosis-related proteins. However, the ectopic effect of terphenyllin on STAT3 and the actual target have not been verified in gastric cancer. Herein, we tested its effect on the STAT3 signaling pathway. We found that terphenyllin inhibited the STAT3 signaling pathway by inhibiting the expression level of p-STAT3 rather than STAT3. Subsequently, we also verified that terphenyllin could reduce the expression levels of proliferation-related protein c-Myc and cell cycle-related protein Cyclin D1, thus inhibiting the proliferation and blocking the cycle progression of gastric cancer cells.

To determine the anticancer effect of terphenyllin *in vitro* and *in vivo*, we used two gastric cancer cells for the cytotoxicity experiment. It showed that terphenyllin has significant cytotoxicity to human gastric cancer cells. We found that terphenyllin inhibited colony formation and cell cycle progression and induced apoptosis in gastric cancer cells in a concentration-dependent manner. Wound healing and transwell invasion experiments showed that terphenyllin had an inhibitory effect on the migration and invasion of gastric cancer cells *in vitro*. Furthermore, we established an orthotopic gastric cancer mouse model, which can provide tumor cells with the same microenvironment *in vivo* as their origin, maintain the original biological characteristics of tumor cells, and better predict the therapeutic effect of test compounds. Our results showed that terphenyllin suppressed tumor growth and metastasis *in vivo*. The results of the body weight and H&E staining showed that terphenyllin did not cause significant weight loss and organ damage in mice, indicating that the terphenyllin is safe at an effective dose.

There are some limitations in our work. In the future, surface plasmon resonance assay may need to be used further to validate the molecular targets of terphenyllin. In addition, we still have not studied the binding sites of terphenyllin on STAT3. We have to explore the deeper mechanism of action like how terphenyllin blocks the phosphorylation of STAT3. And whether the binding of terphenyllin to STAT3 results in anticancer activity should be examined in STAT3 knockdown cells and animals.

In conclusion, this study shows that marine-derived natural product terphenyllin binds to STAT3 inhibits the growth and metastasis of gastric cancer at an effective dose without significant toxicity *in vitro* and *in vivo* by inhibiting the STAT3 signaling pathway. Taken together, the present study provides a promising drug candidate for gastric cancer treatment.

## Data Availability

The raw data supporting the conclusion of this article will be made available by the authors, without undue reservation.
